# Editorial: Photocatalysts for Air Purification: Design, Synthesis, and Mechanism Investigations

**DOI:** 10.3389/fchem.2022.870550

**Published:** 2022-03-16

**Authors:** Pengyu Dong, Fan Dong, Roberto Fiorenza

**Affiliations:** ^1^ Key Laboratory for Advanced Technology in Environmental Protection of Jiangsu Province, Yancheng Institute of Technology, Yancheng, China; ^2^ Research Center for Environmental Science and Technology, Institute of Fundamental and Frontier Sciences, University of Electronic Science and Technology of China, Chengdu, China; ^3^ Department of Chemical Sciences, University of Catania, Catania, Italy

**Keywords:** photocatalytic degradation, air pollutants, photocatalytic mechanism, photocatalytic reactor, VOCs

As air pollution becomes more and more serious in many aspects all over the world, the demand for clean air increases significantly and the development of efficient and environment-friendly air purification technologies is highly encouraged (Kampa and Castanas, 2008; Anwar et al., 2021; Shi et al., 2021; Jbaily et al., 2022).

Among various technologies, photocatalysis is particularly suitable for removing low-concentration air pollutants (e.g., VOCs) at sub-ppm or ppb levels because the conventional adsorption technologies are not very efficient (Boonen and Beeldens, 2014; Yu et al., 2010; Hay et al., 2015; Boyjoo et al., 2017; Ren et al., 2017; Schreck and Niederberger, 2019; Weon et al., 2019). In recent years, much effort has been made to improve the applicability of photocatalytic air purification, such as developing novel photocatalysts for air purification, increasing the optical absorption and charge generation to enhance the photocatalytic activity, and understanding of mechanisms and reactions (Wu and van de Krol, 2012; Dong et al., 2014; Weon and Choi, 2016; Huang et al., 2017; Zhu et al., 2017; Li et al., 2018; Liu et al., 2020; Fiorenza et al., 2022).

Despite the progress made in this field, many issues must be resolved to meet stringent emission standards economically and effectively. Herein, the following aspects are expected to solve problems. 1) Developing efficient photocatalysts with highly exposed reactive facets, abundant defect sites, and strong interfacial interactions to remove the air pollutants, for instance, core-shell-structured materials, hierarchical porous materials, skeleton/channel-confined materials, and single-atom catalytic materials. 2) Designing highly active, universally applicable, and stable photocatalysts with intense resistance to poisons. Indeed, the practical reaction environments are usually very complicated and trace pollutants including water vapor, ammonia, and sulfur-containing compounds may coexist in these streams. 3) Demonstrating how the bond cleavage and oxidation mechanisms of air pollutants are influenced by reaction conditions or times at the molecular level. It can be achieved by applying *in situ* characterization techniques such as FTIR and highly sensitive real-time monitoring techniques such as proton-transfer reaction−mass spectrometry. 4) Establishing how different catalytically active sites (i.e., redox centers, noble metal active sites, and acidic/basic centers) or the application of multi-catalytic approaches, such as photothermal-catalysis, activate the air pollutants and intermediate species to develop a deeper understanding of desirable properties to aid the future design of photocatalysts for purification of air pollutants. 5) Deriving a greater understanding of the deactivation or poisoning mechanisms of different photocatalysts. This can be achieved by establishing correlations between the surface chemistry of photocatalysts and their catalytic performance and exploring effective regeneration methods for deactivated photocatalysts (*in situ* regeneration in particular) to reduce operating costs and ultimately increase industrial viability. 6) Exploiting the developments made in the field of molecular modeling, including the use of theoretical calculations and models to simulate mass and heat transfer effects and predict the reaction behavior of given systems/reactors.

In this research topic, we have invited some scientists worldwide to contribute original research and review articles which could enhance our understanding of some of the above issues in photocatalytic air purification. These original articles have been accepted for publication after peer review. Khan et al. designed a full spectrum-induced hybrid structure consisting of one-dimensional nickel titanate (NiTiO_3_) nanofibers (NFs) decorated with petal-like molybdenum disulfide (MoS_2_) particles, and the key parameters for tailoring the morphology, porosity, surface, and interfacial properties of the photocatalysts were identified. High CO_2_ selectivity was ascribed to the improved light harvesting, the abundance of active edges, insertion of multiphase (2H/1T) MoS_2_, and higher surface area, and partly to the hydrophobic feature of the hybrid structure. He et al. found that the heterojunction photocatalyst containing MoS_2_ and ordered mesoporous carbon (OMC) showed an enhanced photocatalytic efficiency for formaldehyde decomposition and exhibited an excellent regeneration performance after six recycles, indicating the MoS_2_/OMC composite is a promising photocatalyst with high activity and stability for VOCs removal. The excellent regeneration performance was ascribed to the unique structure, in which the hollow spherical MoS_2_ was assembled into an orderly structure of OMC that greatly increased the number of edge active sites and effectively overcame the defect of agglomeration of MoS_2_ nanomaterials. Qian et al. reviewed the breakthroughs and challenges of metal-organic frameworks (MOFs) as powerful photocatalysts for indoor-air VOCs pollutants cleaning (such as aldehydes, aromatics, and short-chain alcohols). It is considered that the active centers of MOFs photocatalysts could be divided into two categories, in which MOFs with variable valence metal nodes act as direct photoactive centers and MOFs with non-variable valence metal nodes but after combining other photoactive variable valence metal centers act as excellent concentrated and concerted electron-transfer materials. Huang et al. designed a photocatalytic reactor of solar updraft towers (SUT) to remove methane from the air at a planetary scale and presented a deep analysis by calculating the potential of methane removal concerning the dimensions and configuration of SUT using various photocatalysts based on the heat transfer mass and transfer models. It is found that the effectiveness of combining photocatalysis with SUT highly depends on the efficacy of photocatalysts (including the catalyst coating area), the size of SUT, and the exploring night operation strategies.

At last, as the Guest Editors of this research topic, we would like to express our gratitude to all the authors for their contributed articles and thank all the referees for their comments on the manuscripts. We hope that the readers will find the results in the articles on this topic interesting and useful for their research. Finally, we appreciate the editorial staff of Frontiers in Chemistry for their work in publishing this research topic.
